# Discharge Performance of Zinc-Air Flow Batteries Under the Effects of Sodium Dodecyl Sulfate and Pluronic F-127

**DOI:** 10.1038/s41598-018-32806-3

**Published:** 2018-10-08

**Authors:** Soraya Hosseini, Woranunt Lao-atiman, Siow Jing Han, Amornchai Arpornwichanop, Tetsu Yonezawa, Soorathep Kheawhom

**Affiliations:** 1 Department of Chemical Engineering, Faculty of Engineering, Chulalongkorn University, Bangkok, 10330 Thailand; 2 Computational Process Engineering Research Unit, Chulalongkorn University, Bangkok, 10330 Thailand; 3 Department of Chemical Engineering, Faculty of Engineering, University Technology PETRONAS, Seri Iskander, Perak, 32610 Malaysia; 4 Division of Materials Science and Engineering, Faculty of Engineering, Hokkaido University, Kita 13 Nishi 8, Sapporo, Hokkaido 060-8628 Japan

## Abstract

Zinc-air batteries are a promising technology for large-scale electricity storage. However, their practical deployment has been hindered by some issues related to corrosion and passivation of the zinc anode in an alkaline electrolyte. In this work, anionic surfactant sodium dodecyl sulfate (SDS) and nonionic surfactant Pluronic F-127 (P127) are examined their applicability to enhance the battery performances. Pristine zinc granules in 7 M KOH, pristine zinc granules in 0–8 mM SDS/7 M KOH, pristine zinc granules in 0–1000 ppm P127/7 M KOH, and SDS coated zinc granules in 7 M KOH were examined. Cyclic voltammograms, potentiodynamic polarization, and electrochemical impedance spectroscopy confirmed that using 0.2 mM SDS or 100 ppm P127 effectively suppressed the anode corrosion and passivation. Nevertheless, direct coating SDS on the zinc anode showed adverse effects because the thick layer of SDS coating acted as a passivating film and blocked the removal of the anode oxidation product from the zinc surface. Furthermore, the performances of the zinc-air flow batteries were studied. Galvanostatic discharge results indicated that the improvement of discharge capacity and energy density could be sought by the introduction of the surfactants to the KOH electrolyte. The enhancement of specific discharge capacity for 30% and 24% was observed in the electrolyte containing 100 ppm P127 and 0.2 mM SDS, respectively.

## Introduction

Renewable energy sources have broadly attracted attention to the global energy supply. Nevertheless, efficient utilization of renewable energy sources requires safe and cost-effective electricity storage systems^1^. Metal-air batteries, consisting of a metal anode coupled with an air-breathing cathode, have received revived interest as they exhibit very high energy density at low cost^2–4^. Specifically, alkaline zinc-air batteries are a promising candidate. Zinc anodes exhibit an electrode potential of −1.26 V vs. standard hydrogen electrode and possess a theoretical specific capacity of 819 mAh/g^5^. Besides, their low-cost, high natural abundance, low toxicity, high safety, high specific energy density and environmental friendliness make them very attractive^6,7^. Recently, zinc-air flow batteries, also known as zinc-air fuel cells, have been demonstrated. These batteries can be quickly refueled with fresh zinc powder or granules^8,9^.

Electrolyte plays an essential role in battery electrochemistry affecting the transport properties of the active species between the anode and the cathode. Also, it determines the energy and power density of the batteries. An enormous research effort has been made to enhance the battery performances by improving the electrolyte as this approach is simple and does not affect the specific energy of the batteries^10,11^.

The ideal electrolyte for zinc-air batteries should exhibit high ionic conductivity with other favorable electrochemical and physicochemical properties, such as nonflammability, low toxicity, large stable potential window, and stability over wide temperature range. Aqueous potassium hydroxide (KOH) solution is extensively used in primary zinc-MnO_2_ cells due to its high solubility of zinc salts and fast electrochemical kinetics^12^. However, there are some limitations in its practical usage in zinc-air batteries due to several disadvantages^13^. The side-reaction of hydrogen evolution, also known as corrosion of the zinc anode, leads to a decrease of discharge capacity. Besides, the accumulation and precipitation of discharged products on the active zinc anode lead to the undesirable passivation effect. Though, neutral aqueous electrolytes demonstrated positive effects of corrosion and carbonate formation issues^14^. However, the low concentration of hydroxide ion (OH^−^) and poor conductivity of neutral electrolytes affect the catalytic activity of MnO_x_ catalysts^15^. Also, the low zincate solubility of neutral electrolytes would make the passivation issue even worse^16^.

The degradation of battery performance, resulting from passivation of the zinc anode in an alkaline electrolyte, is a crucial factor that hinders the practical deployment of the batteries. The passivation effect is known as the result of zinc oxide (ZnO) deposition on the active zinc surface. Zincate ions (${\rm{Zn}}{({\rm{OH}})}_{4}^{2-}$) are formed during the oxidation of zinc. The precipitation of supersaturated zincate ions leads to the formation of Zn(OH)_2_ layers on the zinc surface. Besides, the dehydration of Zn(OH)_2_ leads to the formation of dense zinc oxide passivating layers. Several research works revealed that coating zinc particles with various additives could overcome these issues^17–19^. Aluminum oxide (Al_2_O_3_) as a surface modification exhibited the positive effect on controlling the hydrogen evolution reaction (HER) for zinc-air batteries^20^. Besides, Al_2_O_3_ coating layer prevents direct exposition of zinc to the KOH electrolyte leading to a significant decrease of self-discharge and hydrogen evolution. Also, Schmid *et al*.^21^ studied the electrochemical behavior of zinc particles coated with silicon dioxide (SiO_2_) via chemical solution deposition and zinc silicate (Zn_2_Si_2_O_4_) by chemical vapor deposition.

The application of surfactants, as a corrosion inhibitor, is gaining interest due to their safety and low cost. Surfactants are amphiphilic molecules containing both hydrophobic and hydrophilic groups. Various types of surfactants (anionic, cationic, and nonionic) have been employed in various battery systems to improve their electrochemical performance^21–23^. Corrosion and passivation behaviors of planar zinc was studied in electrolytes containing KOH, ZnO and a cationic surfactant dodecyltrimethylammonium bromide (DTAB)^24^. DTAB exhibited a considerable effect on inhibiting zinc corrosion and passivation. Deyab^25^ investigated nonionic surfactant poly(oxyethylene) nonylphenyl ether as a corrosion inhibitor for zinc corrosion in an alkaline electrolyte. Poly(oxyethylene) nonylphenyl ether is composed of branched 4-nonylphenol and ethylene oxide. The results showed that the surfactant effectively inhibited zinc corrosion. Besides, the application of anionic surfactant sodium dodecyl sulfate (SDS) in a rechargeable Na-Zn battery system was investigated. The corrosion of zinc was effectively suppressed because the adsorbed SDS molecules prevented the contact between water and the anode^26^. Also, anionic surfactant sodium dodecyl benzene sulfonate (SDBS) was studied as an inhibitor for zinc corrosion in NH_4_Cl solution. SDBS exhibited a desirable characteristic for battery application^27^. Yang *et al*.^28^ reported that SDBS significantly suppressed the passivation of the zinc anode in a diluted alkaline electrolyte. Ghavami *et al*.^29^ studied the effects of cationic surfactant cetyl trimethylammonium bromide (CTAB) and SDBS on the performance of Zn–MnO_2_ alkaline batteries. It was reported that the polar group of the surfactant molecule significantly affected the growth of zinc oxide crystal. The negative charge polar group of SDBS coordinated to zinc ions at the anode surface leading to a generation of small zinc oxide particles. In contrary, the positive charge polar group of CTAB showed weak interaction with the zinc ions leading to a generation of larger zinc oxide particles. Furthermore, the effects of SDS and CTAB were investigated on the sulfation of negative active material in lead-acid batteries^30^. The results revealed that SDS outperformed CTAB, and significantly enhanced the cycle life of the batteries.

The primary objective of this work is to examine the effects of SDS, which is an anionic surfactant, and Pluronic F-127 (P127), which is a nonionic surfactant, on the electrochemical behavior of zinc granules used in zinc-air flow batteries. SDS is a common surfactant used in various applications. Its molecule consists of a 12-carbon chain attached to a sulfate group. Its hydrocarbon chain combined with a polar sulfate group give the compound amphiphilic properties. In contrary, P127 is a tri-block copolymer of poly(ethylene oxide)-poly(propylene oxide)-poly(ethylene oxide). Its molecule contains several anchoring groups attached to hydrocarbon chain. P127 has been applied as a corrosion inhibitor in various applications. However, its application in batteries was rarely reported. These two surfactants have different polar groups, and the mechanisms for the adsorption onto the zinc surface are different. Besides, the application of both surfactants in zinc-air flow batteries has not been reported. Thus, in this work, corrosion and passivation behaviors of the zinc granules in 7 M aqueous potassium hydroxide (KOH) electrolyte is studied. The concentration ranges of SDS and P127 investigated are 0–8 mM and 0–1000 ppm, respectively. Besides, direct coating SDS on the pristine zinc granules by soaking in SDS solution is examined. Cyclic voltammetry, electrochemical impedance spectroscopy, potentiodynamic polarization measurements are used to investigate the electrochemical characteristics and mechanisms of zinc oxidation. Also, the performances of the zinc-air flow batteries using these electrolytes were examined and discussed.

## Experimental

### Chemical and Materials

Nickel (Ni) foam with a purity of 99.97%, 100 pores per inch (PPI) and 1 mm thick, purchased from Qijing Trading Co., Ltd., was used as the cathode current collector. The anode current collector and working electrode for cyclic voltammetry analysis were made of 100 mesh of woven wire 304 stainless steel, purchased from Alikafeii Trading Co., Ltd. Pristine zinc granules with a purity of 99.99% and an average diameter of 0.8 mm, purchased from Sirikul Engineering Ltd., Part., were used as the anode. KOH pellets (99%) purchased from CT Chemical Co., Ltd., were used to prepare the electrolytes. Manganese(IV) oxide (MnO_2_, 5 *μ*m 99.99%, Sigma-Aldrich), and carbon black (Vulcan^®^ XC-72, Cabot Corporation) and poly(tetrafluoroethylene) (PTFE powder, 1 *μ*m, Sigma-Aldrich) were used to prepare the cathode. Poly(vinyl butyral) (PVB), purchased from Sigma-Aldrich, was used as a binder. Also, sodium dodecyl sulfate (SDS, 99.8%, Sigma-Aldrich) and Pluronic F-127 (P-127, 99%, Sigma-Aldrich) were used as electrolyte additives. Whatman filter paper No.1 (Sigma-Aldrich) and poly(vinyl acetate) (PVAc) (TOA Paint Public Co., Ltd.) were used to prepare the separator. All chemicals were used as received without any further purification.

### Electrode and battery fabrication

**Figure 1:**
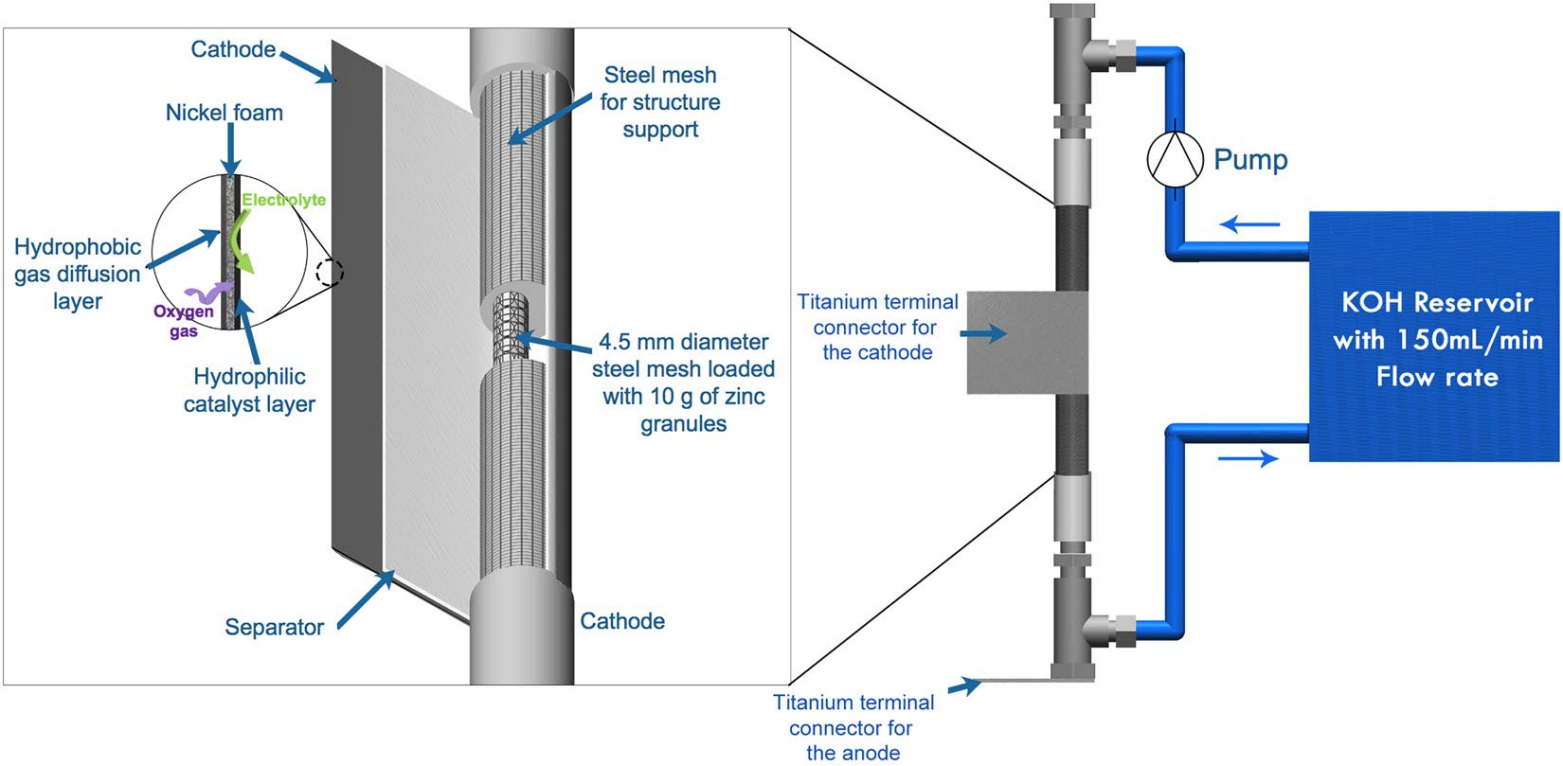
A schematic diagram of the zinc-air flow batteries.

Homemade zinc-air flow batteries were fabricated and used to evaluate polarization and galvanostatic discharge characteristics. The schematic diagram of the batteries is shown in Fig. 1. A stainless steel mesh cylinder with 10.00 cm long and 1.00 cm outer diameter was used as the structural support of the cell. The structural support cylinder was concealed with the separator. The separator was prepared by casting 2 g of 24 wt.% PVAc aqueous solution over both sides of a filter paper and then drying in an oven at 55 °C for 10 min. Afterward, the cylinder was covered with the cathode sheet, consisting of three layers; a gas diffusion layer, a cathode current collector, and a catalyst layer. The catalyst layer is placed in contact with the separator. The active area of the cathode was 20 cm^2^. Nickel foam was employed as the cathode current collector. The catalyst layer was fabricated by casting a slurry mixture of 2 g MnO_2_, 7 g carbon black, 1 g PTFE powder, and 0.45 g PVB in 10 ml ethanol on one side of the nickel foam. Totally 1 g of the slurry was deposited on the nickel foam. The gas diffusion layer was fabricated on the other side of the nickel foam by casting a slurry mixture of 3 g carbon black, 7 g PTFE powder, and 0.5 g PVB in 10 ml of ethanol. Totally 1 g of the slurry was deposited on this side of the nickel foam. The coated nickel foam was then heat-pressed at 350 °C for 5 min using a manual hot press machine. The gas diffusion layer shows hydrophobicity and keeps the electrolyte inside the cell whilst allowing oxygen gas to diffuse to the catalyst layer. Besides, the hydrophobicity of the gas diffusion layer prevents leakage of the electrolyte and water flooding in the cathode.

Another stainless steel mesh cylinder with 0.45 cm outer diameter was employed as the anode current collector. The anode current collector was installed inside the structural support cylinder. The anode made of 10 g pristine zinc granules was loaded inside the anode current collector. For the anode with direct coating SDS layer, pristine zinc granules were soaked into 1 M SDS aqueous solution for 24 hr and then washed several times with ethanol. The coated zinc granules were dried at 60 °C and used as the anode.

KOH aqueous solution (7 M) or KOH aqueous solution (7 M) containing SDS (0–8 mM) or P127 (0–1000 ppm) was used as the electrolyte. The electrolyte with the total volume of 150 mL was fed through the cell at a circulation rate of 150 mL/min using a peristaltic pump.

### Characterization and measurement

Electrochemical impedance spectroscopy (EIS), cyclic voltammetry (CV) and potentiodynamic polarization measurments were carried out in a three-electrode configuration by a potentiostat/galvanostat with impedance measurement unit (AMETEK, PAR VersaSTAT 3A). A platinum plate (20 × 20 mm) was used as the counter electrode. A mercury/mercury oxide (Hg/HgO) electrode was used as the reference electrode. Besides, a stainless steel mesh cylinder with 2.0 mm inner diameter, containing zinc granules with total surface area of 20 mm^2^, was used as the working electrode. After 30 min of immersion of each sample in the solutions at room temperature, the potentiodynamic polarization measurments and EIS were performed. The CV applied with a scan rate of 0.05 V/s (unless otherwise specified) from −1.8 to 0.8 V vs. Hg/HgO was performed under ambient atmosphere. In the forward scan, referred as the anodic trace, the potential was swept from the initial potential of −1.8 V vs. Hg/HgO to the switching potential of 0.8 V vs. Hg/HgO. The scan direction was then reversed, and the potential was swept back to −1.8 V vs. Hg/HgO, referred as the cathodic trace. Also, EIS were performed at the potential 0 V vs. OCV with the frequency range from 1 Hz to 100 kHz with alternate current (AC) amplitude of 5 mV. The potentiodynamic polarization was measured at a scan rate of 0.065 mV/s. Besides, in each experiment, pristine zinc granules were replaced.

In addition, the polarization and galvanostatic discharge characteristics of the batteries were examined using a battery analyzer (Battery Metric, MC2020). The polarization characteristic of the batteries was determined at the electrolyte circulation rate of 150 mL/min. The potentials of the batteries were recorded point-by-point while the discharge current density of the batteries was stepped up from the initial current density of 0 to 100 mA/cm^2^. At each point, the batteries were held for 5 s to allow the system to reach the equilibrium potential. Besides, the galvanostatic discharge profile of the batteries was measured at the electrolyte circulation rate of 150 mL/min. The potential of the batteries was recorded every 20 s while the batteries discharged at a constant current density of 25 mA/cm^2^.

## Results and Discussion

### Cyclic voltammetry

**Figure 2:**
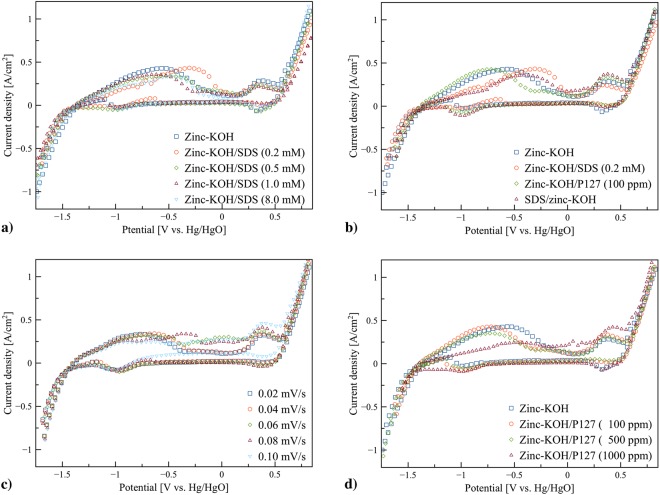
Cyclic voltammograms of pristine zinc granules in KOH electrolyte (zinc-KOH), pristine zinc granules in the KOH-SDS electrolytes (zinc-KOH/SDS), pristine zinc granules in the KOH-P127 electrolytes (zinc-KOH/P127), and SDS coated zinc granules in KOH electrolyte (SDS/zinc-KOH) in the voltage ranging from −1.8 to 0.8 V vs. Hg/HgO using a potential scan rate of 0.05 V/s unless otherwise specified: (**a**) the effects of SDS concentration on voltammetric behavior, (**b**) voltammetric behavior of zinc-KOH, zinc-KOH/SDS (0.2 mM), zinc-KOH/P127 (100 ppm), and SDS/zinc-KOH, (**c**) the effects of scan rate on voltammetric behavior of zinc-KOH/SDS (0.2 mM), and (**d**) the effects of P127 concentration on voltammetric behavior.

The electrochemical characteristics of pristine zinc granules in KOH electrolyte (zinc-KOH), pristine zinc granules in the KOH-SDS electrolytes (zinc-KOH/SDS), pristine zinc granules in the KOH-P127 electrolytes (zinc-KOH/P127), and SDS coated zinc granules in KOH electrolyte (SDS/zinc-KOH) were examined. CV measurements were recorded in the voltage ranging from −1.8 to 0.8 V vs. Hg/HgO using a potential scan rate of 0.05 V/s unless otherwise specified. The cyclic voltammograms of zinc-KOH, zinc-KOH/SDS, zinc-KOH/P127 and SDS/zinc-KOH are shown in Fig. 2. Similar cyclic voltammograms with two anodic peaks were obtained for all cases. The onset potential for oxygen evolution was at 0.5 V vs. Hg/HgO whilst hydrogen evolution took place below −1.4 V vs. Hg/HgO. The onset potential of zinc dissolution appeared at −1.4 V vs. Hg/HgO on the forward scan. The peaks associated with zinc dissolution were observed between −0.6 and −0.3 V vs. Hg/HgO. The peaks at around 0.3 V vs. Hg/HgO are related to the oxidation of Chromium (Cr) on the surface of stainless steel mesh^31^. The cyclic voltammogram of stainless steel mesh without zinc granules is shown in Fig. S1. The voltammograms revealed the oxidation peaks around 0.3 V vs. Hg/HgO which are similar to the previous report^31^.

Figure 2a shows the cyclic voltammograms of zinc-KOH/SDS as a function of SDS concentration. The presence of 0.2 mM SDS increased the peak current density and the peak area but shifting the peak towards positive potentials. The increase of the peak area represents the increase of zinc dissolution. Nevertheless, adding a higher amount of SDS, the peak area decreased indicating the decrease of zinc dissolution. The cyclic voltammograms of zinc-KOH/SDS for 0.2, 0.5 and 1 mM (below critical micelle concentration (CMC) of SDS which is around 7.7 mM) and 8 mM (above CMC of SDS) show a similar shape and peak potential. The optimum amount of SDS was 0.2 mM with the highest zinc dissolution.

Figure 2b displays the comparison between the cyclic voltammograms of zinc-KOH, zinc-KOH/P127, zinc-KOH/SDS and SDS/Zinc-KOH. The results revealed a significant difference in the location of the peak for zinc dissolution. By adding SDS to KOH solution, the peak of zinc dissolution occurred in more positive potentials. In contrast, using P127 as an additive, the peak of zinc dissolution appeared in more negative potentials. Thus, adding of SDS to KOH solution is more effective in inhibiting the corrosion of zinc anode than that of P127. Besides, the results showed that the peak area related to zinc dissolution in SDS/zinc-KOH is smaller than that of zinc-KOH. In this case, thick layers of SDS directly adsorbed on the zinc surface formed a passivating layer. The SDS layers are very effective in suppressing the dissolution of zinc and also preventing a transfer of discharged products from the zinc surface to the electrolyte. Therefore, the migration of zincate ion towards the electrolyte and the transfer of hydroxide ion (OH^−^) from the electrolyte to the surface of the anode are impeded resulting in the formation of zinc oxide directly on the active zinc surface. The accumulation of the discharged products further accelerated the formation of zinc oxide layer inhibiting the zinc dissolution process.

Figure 2c shows the effect of scan rate 0.01 to 0.1 mV/s for the sample containing 0.2 mM SDS. The magnitude and area of the oxidation peaks slightly changed with the scan rate. By increasing the scan rate from 0.01 to 0.05 mV/s, the peak current density remained constant but shifted towards more positives potentials. However, an increase in the scan rate from 0.06 to 0.1 mV/s, two anodic peaks were merged into a single peak. The results suggested that in this system, the mechanism of zinc dissolution is rather complex. As the working electrode is made of zinc granules loaded inside the stainless steel mesh cylinder, the contact resistance between zinc granule and zinc granule, and zinc granule and the current collector is significant and varied. Also, the resistance depends on the oxide layers formed on the surface of the zinc granules. By increasing the scan rate, the contact resistance also increased because of the increasing of the oxide product resulting in the shift of the peak towards more positives potentials. The effects of the scan rate in the system using a zinc plate instead of zinc granules/stainless steel mesh cylinder were also examined. The results are shown in Fig. S2. The onset potential of zinc dissolution occurred around −1.4 V vs. Hg/HgO on the forward scan. Besides, the peak current is proportional to the square root of the scan rate suggesting that the zinc dissolution is in a diffusion regime. Also, the results suggested that the shape and configuration of the anode plays a significant role in the mechanism of zinc dissolution.

Figure 2d shows the effects of P127 concentration on the cyclic voltammograms of zinc-KOH/P127. In all cases, two anodic peaks of zinc dissolution and Cr oxidation were observed. The highest peak area of zinc oxidation was observed at 100 ppm P127. At higher concentration of P127, the lower zinc dissolution compared to that of zinc-KOH was observed. The critical micelle concentration (CMC) of P127 is around 995 ppm. At the concentration lower than CMC, the surfactant molecules tend to adsorb on exposed interfaces in which surface tension reduces due to interfacial aggregation. At the concentration higher than CMC, layers of surfactant molecules are deposited on the metal surface, and also free micelles are formed in the electrolyte. Thus, the passive layer thickness can limit the diffusion of hydroxide ion to the metal surface.

### Potentiodynamic polarization measurement

**Figure 3:**
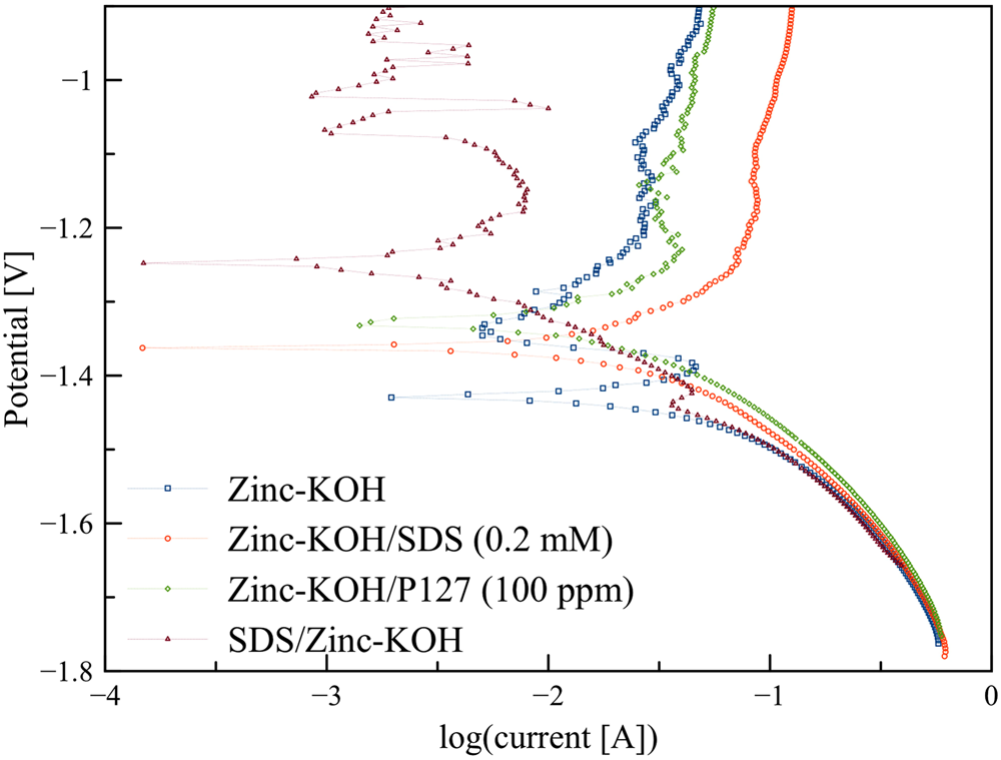
Potentiodynamic polarization measurement using a scan rate 0.065 mV/s in the range −0.5 to 0.5 V (vs. OCV).

The adsorption of surfactants on the metal surface can noticeably change its corrosion inhibition property. The corrosion behavior of zinc anode influences the performances and shelf-life of the batteries. Therefore, the potentiodynamic polarization test was performed to evaluate the corrosion behavior. The experiments were carried out with a scan rate 0.065 mV/s in the different electrolytes within the potential range from −0.5 to 0.5 V vs. OCV. Tafel plots of zinc-KOH, zinc-KOH/SDS, zinc-KOH/P127 and SDS/zinc-KOH are shown in Fig. 3.

Potentiodynamic polarization curves (Tafel curves) of zinc-KOH/SDS, zinc-KOH/P127 and SDS/zinc-KOH were shifted to more positive voltage region compared to that of zinc-KOH, suggesting that the surfactants reduced the rate of the anodic reaction. Therefore, the surfactants could effectively inhibit the corrosion tendency of the zinc anode. SDS coated zinc revealed more shifting of potential that can be ascribed to the protective film of SDS over the zinc active surface protecting the surface against KOH solution. The rest potential of SDS/zinc-KOH is −1.25 V whilst zinc-KOH exhibited a more negative potential of −1.43 V suggesting that SDS coatings could effectively inhibit the corrosion of pristine zinc granules. Besides, the rest potentials of zinc-KOH/SDS (0.2 mM) and zinc-KOH/P127 (100 ppm) are −1.36 V and −1.33 V, respectively. The more negative of the rest potential indicates more sensitive of the material to the corrosion. Therefore, adding SDS or P127 could enhance anti-corrosion performance.

The cathodic and anodic branches of the corresponding Tafel curves are asymmetry, suggesting an irreversible electrochemical reaction. Besides, a strong active-passive behavior was observed in zinc-KOH and SDS/Zinc-KOH. As the potential increased above the rest potential, the current density exponentially increased as expected for an active dissolution of the zinc anode. Nevertheless, at the potential called the Flade potential^32^, the trend of the current density reversed and decreased by many orders of magnitude due to the localized formation of passivation films. In the passive region, the current density was independent of the potential. In comparison with an extrapolation of the current behavior in the active region, the current density in the passive region decreased enormously. However, at a higher potential, the current density increased again because of the breakdown of the passivation films.

The Tafel equations for the anodic and cathodic reactions can be described by the Butler-Volmer equation as shown in (1) ^33^.

1 $$I={I}_{{\rm{corr}}}({e}^{\tfrac{\mathrm{2.303(}E-{E}_{{\rm{corr}}})}{{\beta }_{a}}}-{e}^{\tfrac{\mathrm{2.303(}E-{E}_{{\rm{corr}}})}{{\beta }_{c}}})$$

*I* is the measured current. ${I}_{{\rm{corr}}}$ is the corrosion current. *E* is the electrode potential. ${E}_{{\rm{corr}}}$ is the corrosion potential also known as the rest potential. *β*_*a*_ and *β*_*c*_ are the Tafel slopes of the anodic and cathodic partial reactions.

At the potentials close to ${E}_{{\rm{corr}}}$, the Tafel curves can be approximated as a straight line. The slope of this line is called the polarization resistance (${R}_{p}$). ${R}_{p}$ can be determined using the Stern-Geary equation^34^ as shown in (2), and it has the unit of resistant. ${R}_{p}$ is the transition resistance between the electrodes and the electrolyte. An electrode is polarized when its potential is forced away from the rest potential. Polarization of an electrode causes current to flow due to electrochemical reactions at the electrode surface.

2 $${R}_{p}=\frac{{\beta }_{a}|{\beta }_{c}|}{2.303{I}_{{\rm{corr}}}({\beta }_{a}+|{\beta }_{c}|)}$$

Table 1 displays all corrosion and electrochemical parameters obtained from Tafel fitting of potentiodynamic polarization measurement. The corrosion current indicates the corrosion tendency of the zinc anode. The higher corrosion current implies higher corrosion tendency of the anode. The corrosion currents of zinc-KOH, zinc-KOH/SDS (0.2 mM), zinc-KOH/P127 (100 ppm), and SDS/zinc-KOH are 15.54, 10.51, 10.19 and 3.51 mA, respectively. The results agreed with the corrosion potentials. Furthermore, the polarization resistance can be used to determine the anti-corrosion property of the anode. The corrosion rate is related to the polarization resistance. The higher polarization resistance means lower corrosion current. Therefore, the higher polarization resistance indicates higher corrosion inhibition property. In addition, the anodic Tafel slope (${\beta }_{a}$) indicates the corrosion tendency of the anode. The Tafel slope depends on the reaction mechanism. Also, the Tafel slope indicates how much overpotential is required to increase the reaction rate. The smaller slope indicates higher corrosion of zinc (dissolution of zinc as well) in KOH solution. The largest anodic Tafel slope occurred in SDS/zinc-KOH whilst zinc-KOH exhibited the smallest anodic Tafel slope. Therefore, the surfactants could suppress corrosion of the anode.

**Table 1:** Corrosion and electrochemical parameters obtained from Tafel fitting of potentiodynamic polarization measurement using a scan rate 0.065 mV/s in the range −0.5 to 0.5 V (vs. OCV).

	zinc-KOH	zinc-KOH/SDS	zinc-KOH/P127	SDS/zinc-KOH
E_corr_ (V)	−1.43	−1.36	−1.33	−1.25
I_corr_ (mA)	15.54	10.51	10.19	3.51
*β*_*a*_ (mV/dec)	74.08	106.20	132.41	198.41
*β*_*c*_ (mV/dec)	−63.41	−77.31	−147.50	−91.72
*R*_*p*_ $({\rm{\Omega }})$	0.95	2.17	2.97	7.75

### Electrochemical Impedance spectroscopy

**Figure 4:**
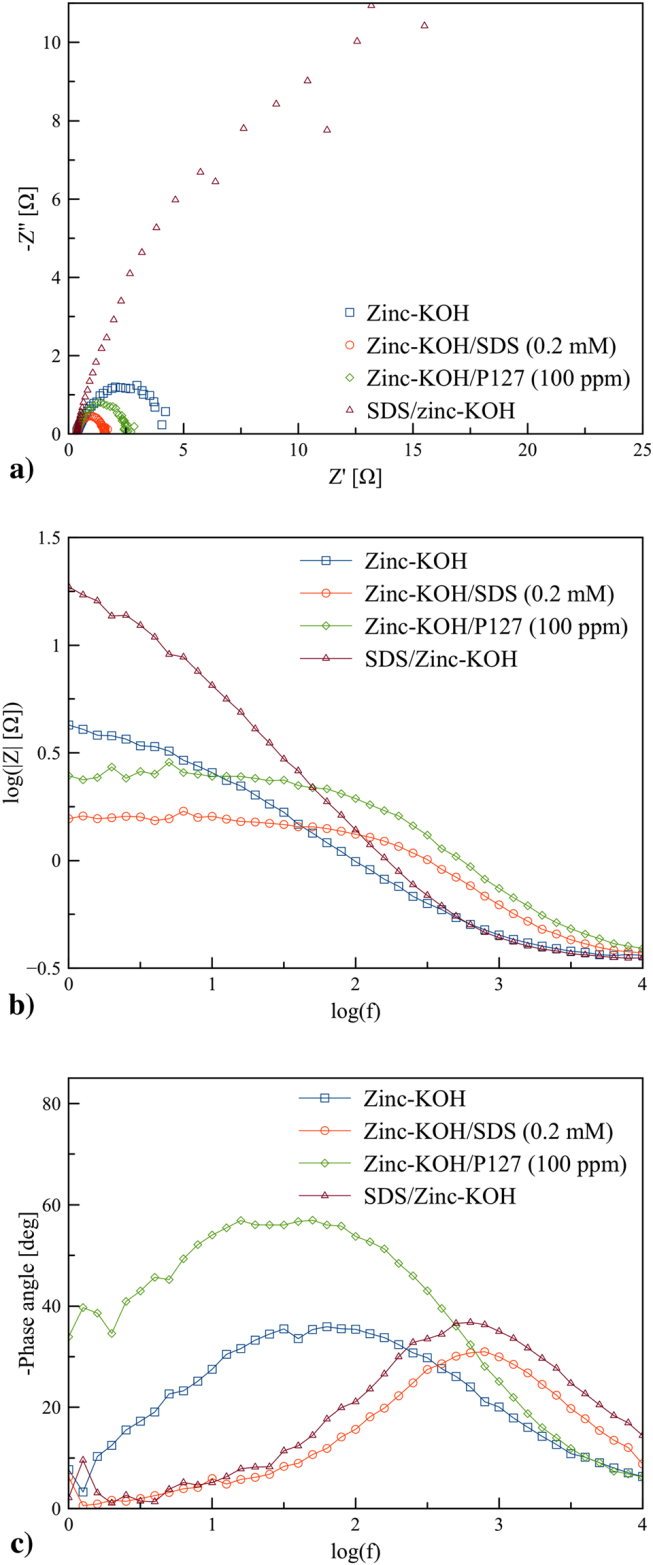
Electrochemical Impedance spectroscopy performed at the potential 0 V (vs. OCV) in the frequency range 1 Hz to 100 kHz with AC amplitude of 5 mV: (**a**) Nyquist Plot, (**b**) Bode plot of log|*Z*| vs. log(*f*), and (**c**) Bode plot of the phase angle vs. log(*f*).

EIS was carried out on pristine zinc granules, and SDS coated zinc granules in various electrolytes using frequency ranging from 1 Hz to 100 kHz with AC amplitude of 5.0 mV. EIS measurements were used to examine the fundamental behavior and to compare the charge transport kinetics of the zinc anode. The Nyquist and Bode plots of impedance spectra of zinc-KOH, zinc-KOH/SDS, zinc-KOH/P127 and SDS/zinc-KOH are presented in Fig. 4. The results showed semicircles, also known as depressed capacitive loops, indicating that the charge transfer process was involved at the electrode/solution interface. The difference of the real part of impedance at the highest and the lowest frequencies indicates the charge transfer resistance (${R}_{{\rm{ct}}}$). The Nyquist plots showed similar shapes in all cases indicating that there is no change in the mechanism of zinc dissolution. Zinc-KOH/SDS (0.2 mM) and zinc-KOH/P127 (100 ppm) exhibited a significant decrease in charge-transfer resistance through the adsorption of the zinc anode oxidation product on the surfactant molecules thus preventing direct precipitation of zinc oxide on the active zinc surface. The radius of the semicircles was influenced by the concentration and the structure of the surfactant.

As presented in Fig. 4a, the non-perfect semi-circle of Nyquist plot in SDS/zinc-KOH was attributed from the inhomogeneity as well as repeated formation and breakdown of passivation films of the anode surface. By using the surfactants, the electrolyte showed an increase in the semi-circular diameter of the capacitance loop, whereas SDS coated zinc had an increase in the semicircular diameter even higher than that of the pristine zinc granules. The surfactants enhanced the zincate ion transfer rate by coordinating and transporting the complexes to the bulk solution. Thus, the higher zincate ion formation and lesser zinc oxide precipitation resulted from the surfactants. Besides, the Bode plots of log |*Z*| as a function of log(*f*) (Fig. 4b) and the phase angle as a function of log(*f*) (Fig. 4c) can be used to study the variation in impedance as a function of frequency.

The low-frequency region of the Bode plots represents the charge transfer resistance (${R}_{{\rm{ct}}}$). Zinc-KOH indicated the lowest charge resistance indicating the fast kinetics of charge transfer reactions associated with the redox process. Zinc-KOH/SDS (0.2 mM) and zinc-KOH/P127 (100 ppm) exhibited higher charge transfer resistance indicating the surface of the anode was occupied by surfactant molecules. The highest resistance was appeared for SDS/zinc-KOH due to a passive layer on the anode surface. The results suggested that the electrolyte containing SDS or P127 exhibited lower impedance compared to SDS/zinc-KOH. Also, the result was consistent with the result of CV and potentiodynamic polarization measurements.

In Fig. 4c, the distinct difference of the impedance modulus was not observed, and the trend of the impedance modulus was similar to that of the charge transfer resistance. Also, a single peak for each sample was observed. The single peak corresponds to a single loop in Fig. 4a. The results indicated the existence of one time constant in its equivalent circuit. The increase of phase angle at relaxation frequency occurred with the order SDS/zinc-KOH > zinc-KOH/P127 > zinc-KOH/SDS > zinc-KOH indicating the increase of capacitive response with that order.

**Figure 5:**
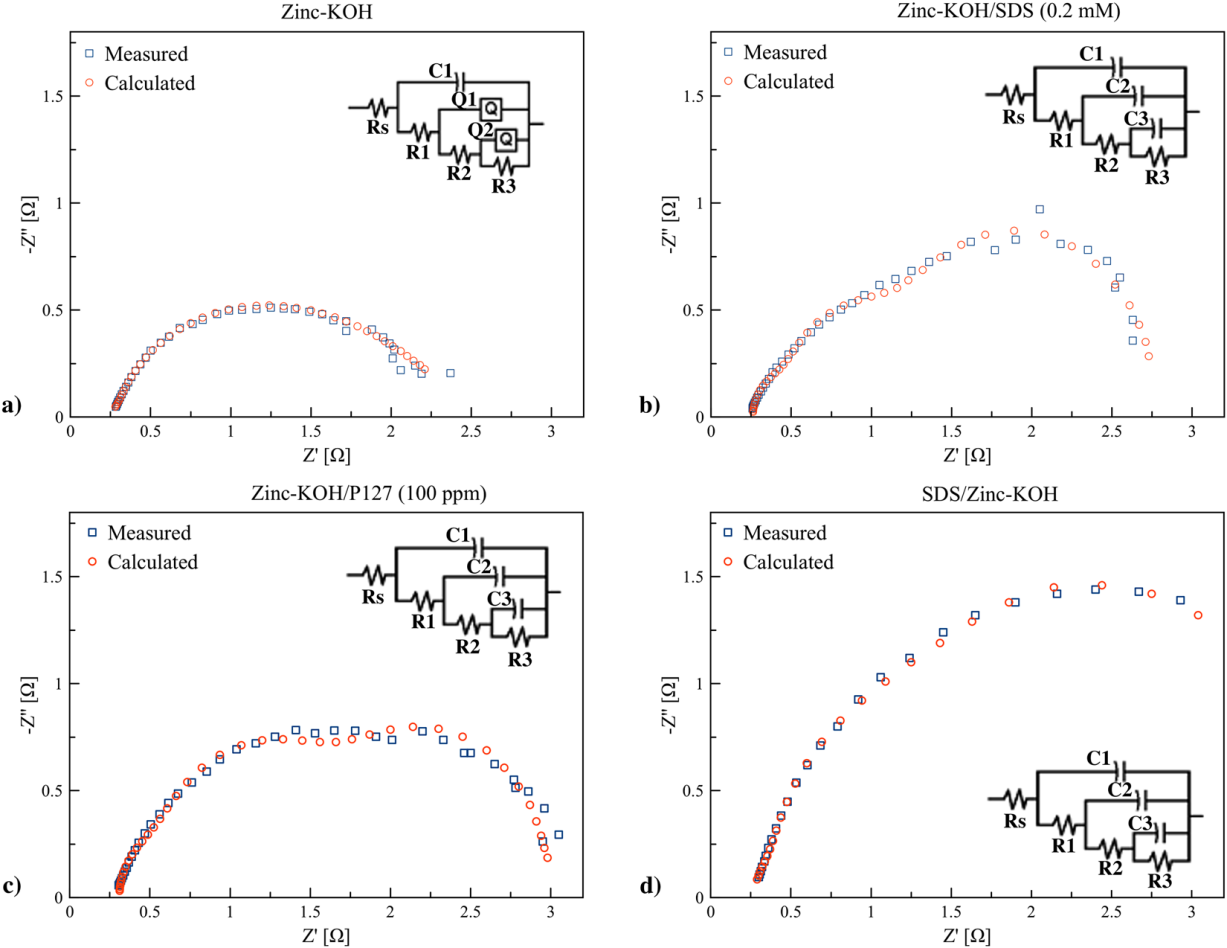
Nyquist Plot of EIS performed at the potential 0 V (vs. OCV) in the frequency range 1 Hz to 100 kHz with AC amplitude of 5 mV, and the simulation using equivalent circuit models: (**a**) zinc-KOH, (**b**) zinc-KOH/SDS (0.2 mM), (**c**) zinc-KOH/P127 (100 ppm), and (**d**) SDS/zinc-KOH.

The impedance spectra of different Nyquist plots were analyzed by fitting the experimental data to simple equivalent circuit models which shown in Fig. 5. The models include resistant *R* and constant phase element (CPE, *Q*). The EIS data fitted by Zsimpwin software are presented in Fig. 5. CPE was used in place of the capacitor in the electrochemical process to compensate for the deviation from ideal dielectric behavior. The non-ideal capacitance response may arise from the inhomogeneous nature of the electrode surface^35^. The CPE is composed of a component *Q* and coefficient *n*. It can be attributed to various physical phenomena such as surface roughness, porous layer formation, and inhibitor adsorption. The double layer capacitance (*C*_*dl*_) can be estimated using (3).

3 $${C}_{dl}=Q{\mathrm{(2}\pi {f}_{{\rm{\max }}})}^{n-1}$$

*f*_max_ represents the frequency at the maximum value of imaginary value on the Nyquist plot^36^. In addition to the charge transfer resistance, there are additional resistance components, created via the film resistance, diffuse layer resistance, and the resistance due to accumulations of inhibitor molecules or corrosion products. Herein, the additional resistance may be assigned for an outer porous zinc oxide layer. The parameters of the equivalent circuit, used for simulations, are shown in Table 2.

**Table 2:** EIS parameters obtained by fitting the data to equivalent circuit models.

	Zinc-KOH		Zinc-KOH/SDS		Zinc-KOH/P127		SDS/zinc-KOH
${R}_{{\rm{s}}}[{\rm{\Omega }}]$	0.255	${R}_{{\rm{s}}}[{\rm{\Omega }}]$	0.260	${R}_{{\rm{s}}}[{\rm{\Omega }}]$	0.380	${R}_{{\rm{s}}}[{\rm{\Omega }}]$	0.271
*C*_1_ [F]	2.140 × 10^−4^	*C*_1_ [F]	6.777 × 10^−4^	*C*_1_ [F]	4.989 × 10^−4^	*C*_1_ [F]	1.718 × 10^−4^
${R}_{{\rm{1}}}[{\rm{\Omega }}]$	0.093	${R}_{{\rm{1}}}[{\rm{\Omega }}]$	0.039	${R}_{{\rm{1}}}[{\rm{\Omega }}]$	0.558	${R}_{{\rm{1}}}[{\rm{\Omega }}]$	0.396
*Q*_1_ [Ssec^n^]	3.644 × 10^−4^	*C*_2_ [F]	2.114 × 10^−3^	*C*_2_ [F]	1.213 × 10^−3^	*C*_2_ [F]	2.217 × 10^−4^
*n*	1						
*C*_dl_ [F]	3.644 × 10^−4^						
${R}_{{\rm{2}}}[{\rm{\Omega }}]$	0.047	${R}_{{\rm{2}}}[{\rm{\Omega }}]$	0.864	${R}_{{\rm{2}}}[{\rm{\Omega }}]$	1.109	${R}_{{\rm{2}}}[{\rm{\Omega }}]$	1.638
*Q*_2_ [Ssec^n^]	3.882 × 10^−2^	*C*_3_ [F]	1.430 × 10^−2^	*C*_3_ [F]	1.417 × 10^−2^	*C*_3_ [F]	1.213 × 10^−3^
*n*	0.47						
*C*_dl_ [F]	7.239 × 10^−1^						
${R}_{{\rm{3}}}[{\rm{\Omega }}]$	2.103	${R}_{{\rm{3}}}[{\rm{\Omega }}]$	1.268	${R}_{{\rm{3}}}[{\rm{\Omega }}]$	1.022	${R}_{{\rm{2}}}[{\rm{\Omega }}]$	1.586

The impedance spectra were fitted using an equivalent circuit consisting of the resistance electrolyte (*R*_s_), solid electrolyte interface, charge transfer, capacitance and constant phase element. The electrolyte resistance (*R*_s_) for zinc-KOH (0.255 ${\rm{\Omega }}$), zinc-KOH/SDS (0.260 ${\rm{\Omega }}$) and SDS/zinc-KOH (0.271 ${\rm{\Omega }}$) are almost similar. However, zinc-KOH/P127 exhibits the highest value (0.380 ${\rm{\Omega }}$). A slight increase of *R*_s_ was observed by adding the surfactants to the KOH electrolyte. The small increase of *R*_s_ for the samples containing the surfactants resulted from the change of chemical and physical properties of the electrolyte. The electrolyte/electrode interface resistance of SDS/zinc-KOH was significantly large compared to the others, indicating that thick SDS layer functions as a passivation film with high resistance. Also, zinc-KOH exhibited the double layer capacitance (*C*_dl_) of 3.644 × 10^−4^ F and 7.239 × 10^−1^ F. This double layer is formed as ions and zinc oxide molecules adsorbed onto the anode surface. In comparison, the presence of surfactants could prevent the precipitation of zinc oxide on the anode, and thus improve the charge transfer. Therefore, the behavior of double layer capacitance was not observed.

### Battery performance

**Figure 6:**
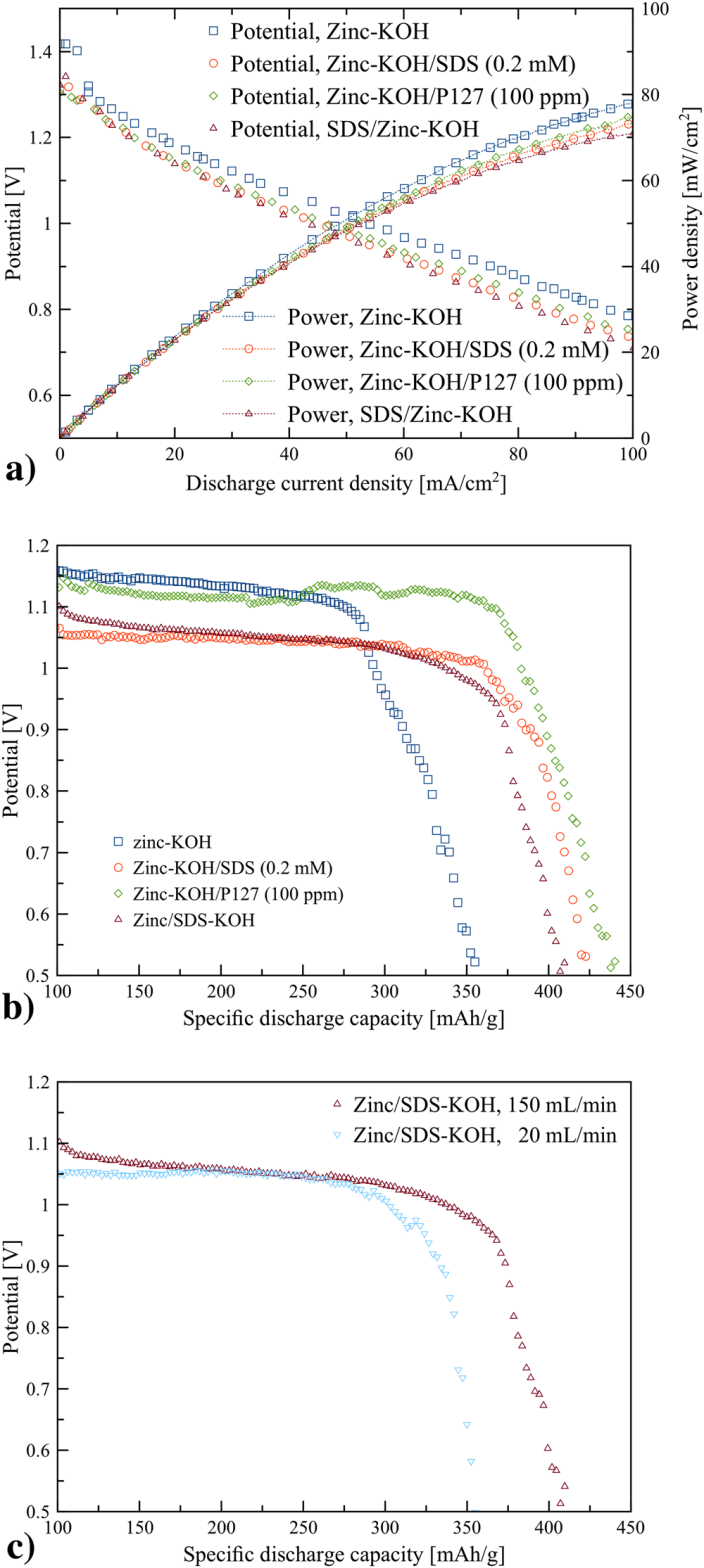
Performance of the zinc-air flow batteries: (**a**) polarization characteristics of the batteries with the electrolyte circulation rate of 150 mL/min, (**b**) galvanostatic discharge profiles of the batteries with the electrolyte circulation rate of 150 mL/min at the discharge current density of 25 mA/cm^2^ and (**c**) galvanostatic discharge profiles of the batteries using SDS/zinc-KOH with the electrolyte circulation rates of 20 and 150 mL/min at the discharge current density of 25 mA/cm^2^.

Electrochemical performances of zinc-KOH, zinc-KOH/SDS, zinc-KOH/P127 and SDS/zinc-KOH were examined using the zinc-air flow batteries operated at the electrolyte circulation rate of 150 mL/min. Polarization characteristics and galvanostatic discharge profiles of pristine zinc in KOH electrolyte (zinc-KOH), pristine zinc in the KOH-SDS electrolytes (zinc-KOH/SDS), pristine zinc in the KOH/P127 electrolytes (zinc-KOH/P127) and SDS coated zinc in KOH electrolyte (SDS/zinc-KOH) are shown in Fig. 6. The power density and voltage of the batteries are a function of the discharge current density as shown in Fig. 6a. All samples revealed a linear voltage drop with an increase of the discharge current density, indicating that the ohmic losses dominated the cell performance. Also, the surfactants exhibited minimal effects on polarization characteristics of the batteries. SDS/zinc-KOH demonstrated the lowest cell performance. At 100 mA/cm^2^ discharge current density, zinc-KOH, zinc-KOH/SDS, zinc-KOH/P127 and SDS/zinc-KOH exhibited (0.78 V, 78 mW/cm^2^), (0.75 V, 75 mW/cm^2^), (0.74 V, 74 mW/cm^2^) and (0.71 V, 71 mW/cm^2^), respectively.

Figure 6b shows the discharge profiles of the batteries at the discharge current density of 25 mA/cm^2^. The profile in each case is similar to typical discharge profiles of zinc-air batteries using a zinc plate as the anode^37^ or porous zinc^38^. Zinc-KOH/P127 (100 ppm) exhibited the highest discharge capacity of 380 mAh/g and 430 mWh/g at a cut-off voltage of 1.0 V. Zinc-KOH/SDS, zinc-KOH/P127 and SDS/zinc-KOH exhibited a stable flat plateau of discharge voltage for a longer discharge period, confirming the positive effect of surfactants on the discharge performance. In contrast, zinc-KOH yielded the lowest specific capacity of 290 mAh/g (330 mWh/g) at a cut-off voltage of 1.0 V. That is, a drop of 90 mAh/g (100 mWh/g) was observed between zinc-KOH and zinc-KOH/P127. In other words, by adding 100 ppm of P127 to the KOH electrolyte, an improvement of 30% for the specific capacity was observed. Both SDS/zinc-KOH and zinc-SDS/KOH exhibited 350 mAh/g (360 mWh/g) and 360 mAh/g (385 mWh/g) at a cut-off voltage of 1.0 V, respectively. However, these discharge curves are in the lower voltage.

During discharge, the zinc granules is oxidized to form zincate ion^20^. Consequently, saturated zincate ion can precipitate as solid zinc oxide on the active zinc surface.

4 $${\rm{Zn}}+4{{\rm{OH}}}^{-}\to {\rm{Zn}}{({\rm{OH}})}_{4}^{2-}+2{{\rm{e}}}^{-}$$

5 $${\rm{Zn}}{({\rm{OH}})}_{4}^{2-}\leftrightarrow {\rm{ZnO}}+2{{\rm{OH}}}^{-}+{{\rm{H}}}_{2}{\rm{O}}$$

When the zincate concentration exceeds the local solubility limit, zinc oxide will precipitate on the surface of the zinc. The precipitated layer is loose and porous, and does not fully passivate or block the active zinc surface. Nevertheless, it affects ion transport between the electrolyte and zinc surface, impeding hydroxide and zincate ions transport^23,39^. The addition of surfactants to KOH solution prevents zinc oxide precipitation on the active zinc surface, and thus improves the discharge capacity of the batteries. However, P127 revealed the higher voltage in compared to SDS. It may result from sodium ion (Na^+^) in SDS molecule, negatively affecting the diffusivity of hydroxide and zincate ions. Moreover, SDS and P127 contain different types of anchoring groups, and therefore the mechanisms for the adsorption of the surfactants onto the zinc surface are different.

The zinc oxide formation is multi-stage and complex process. Zincate ion is formed through the reaction between zinc and hydroxide ion and subsequently transformed to zinc oxide. In this step, small molecules of zinc oxide can collide with each other and produce a larger group. Stable zinc oxide crystals are obtained when the critical size has been reached.

P127 as a nonionic surfactant has multiple hydrophobic and hydrophilic sections. In addition to the adsorption of P127 on the zinc surface, P127 tends to adsorb on the interface of colloidal particles resulting in stabilization of zinc oxide dispersion. The mechanism of zinc oxide formation in the presence of SDS as an anionic surfactant may be similar to nonionic surfactant. However, SDS molecule contains a single hydrocarbon chain combined with a single polar sulfate group possessing the amphiphilic properties. Thus, the multiple anchoring groups per molecule of P127 provide superior interaction, and strong adsorption with substrates. In contrary, SDS posses a single anchoring group and showed weaker surface adsorption. Also, the hydrogel formation of P127 molecule minimized sedimentation of zinc oxide precipitation.

Besides, it is worth to discuss the results of zinc-KOH/SDS and SDS/zinc-KOH. Though CV, potentiodynamic polarization, and EIS indicated adverse effects of thick SDS coating layers in SDS/zinc-KOH, the discharge capacity of SDS/zinc-KOH was slightly lower than that of zinc-KOH/SDS. It may result from the effects of flowing electrolyte stream in the flow batteries. The flowing electrolyte stream reduces the effects of a passivation layer by removing loosely deposited passivation layers, and reducing local concentration gradients. Figure 6c confirms the effects of the flowing electrolyte stream. At the electrolyte circulation rate of 20 mL/min, the discharge capacity of SDS/zinc-KOH significantly decreased. The specific discharge capacity of 300 mAh/g (315 mWh/g) at a cut-off voltage of 1.0 V was observed. Nevertheless, at the electrolyte circulation rate of 150 mL/min, SDS/zinc-KOH exhibited 350 mAh/g (360 mWh/g) at a cut-off voltage of 1.0 V. That is, by increasing the electrolyte circulation rate from 20 mL/min to 150 mL/min, an improvement of 16% for the specific capacity was observed.

Furthermore, the performances of the batteries are compared with other zinc-air batteries reported in the literature^40,41^ as shown in Table 3. Though, the direct comparison cannot be easily made as these batteries employ different configuration, different catalyst, different shape of zinc. The flow batteries, used in this work, exhibited high discharge capacity.

**Table 3:** Comparison of zinc-air batteries previously reported.

Anode	zinc foil	zinc foil	zinc granules
Catalyst	NiFe/C	NiCo_2_O_4_ spinel	MnO_2_/C
Open circuit voltage (V)	1.3	1.42	1.4
Current density (mA/cm^2^)	100	150	25
Discharge capacity (mAh/g)	140	50	400
Electrolyte	30 wt.% + ZnO	6 M KOH + 0.5 M ZnO	7 M KOH + surfactant
Reference	Wang *et al*.^40^	Pichler *et al*.^41^	This work

In addition, the battery using the electrolyte containing 100 ppm P127 was tested for multiple discharge cycles. For each cycle, 10 g of zinc granules were loaded. The battery discharged at the constant discharge current density of 25 mA/cm^2^ with the electrolyte circulation rate of 150 mL/min. Once all the zinc granules were consumed entirely, the electrolyte was then replaced. Then, 10 g of fresh zinc granules were refilled. The battery repeatedly discharged at the same condition. The results are shown in Fig. S3. The discharge profile in each cycle shows a similar characteristic. The results confirmed that the developed zinc-air flow batteries are refuellable.

## Conclusion

Addition of anionic surfactant Sodium Dodecyl Sulfate (SDS) and nonionic surfactant Pluronic F-127 (P127) to 7M KOH alkaline electrolyte was found to improve the performance of the zinc-air flow batteries by suppressing corrosion and passivation of the zinc anode. The surfactants adsorb zinc oxide molecules and prevent the formation of compact zinc oxide layers which completely passivating the active zinc anode. The results were confirmed using cyclic voltammetry, electrochemical impedance spectroscopy, potentiodynamic polarization measurements. The improvement of the specific capacity for 30% was obtained using 100 ppm P127. In comparison, the improvement of the specific capacity for 24% was obtained using SDS 0.2 mM. Adding the surfactant to the alkaline electrolyte is a simple but effective way to improve performances of the zinc-air flow batteries. Besides, this approach can be applied in other metal-air batteries suffering anode corrosion and passivation.

## Electronic supplementary material

Supplemental information

## Data Availability

The authors declare that all relevant data are within the paper.
