# Supplementary material for: Discharge Performance of Zinc-Air Flow Batteries Under the Effects of Sodium Dodecyl Sulfate and Pluronic F-127
Source: Sci Rep. 2018 Oct 8;8:14909. doi: 10.1038/s41598-018-32806-3 (PMC6175836; doi:10.1038/s41598-018-32806-3)
Supplement: Supplementary file 1 — Supplemental information [file 41598_2018_32806_MOESM1_ESM.pdf]

Discharge Performance of Zinc-Air Flow Batteries Under the Effects of Sodium Dodecyl Sulfate and Pluronic F-127

Soraya Hosseini[1], Woranunt Lao-atiman[1], Siow Jing Han[1,3], Amornchai Arpornwichanop[1,2], Tetsu Yonezawa[4], and Soorathep Kheawhom[1,2,*]

[1]Department of Chemical Engineering, Faculty of Engineering, Chulalongkorn University, Bangkok 10330, Thailand
[2]Computational Process Engineering Research Unit, Chulalongkorn University, Bangkok 10330, Thailand
[3]Department of Chemical Engineering, Faculty of Engineering, University Technology PETRONAS, Seri Iskander,Perak 32610, Malaysia
[4]Division of Materials Science and Engineering, Faculty of Engineering, Hokkaido University, Kita 13 Nishi 8, Sapporo, Hokkaido, 060-8628, Japan

[*]soorathep.k@chula.ac.th

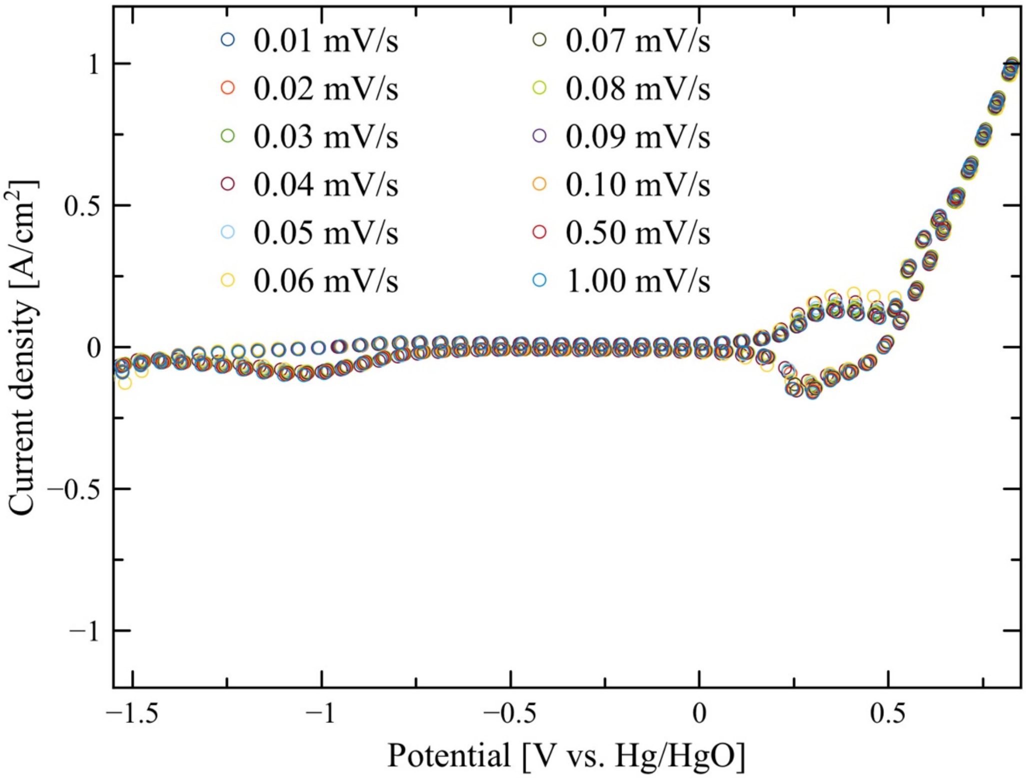

Fig. S1 The effects of scan rate on the voltammetric behavior of a stainless steel mesh cylinder in KOH electrolyte.

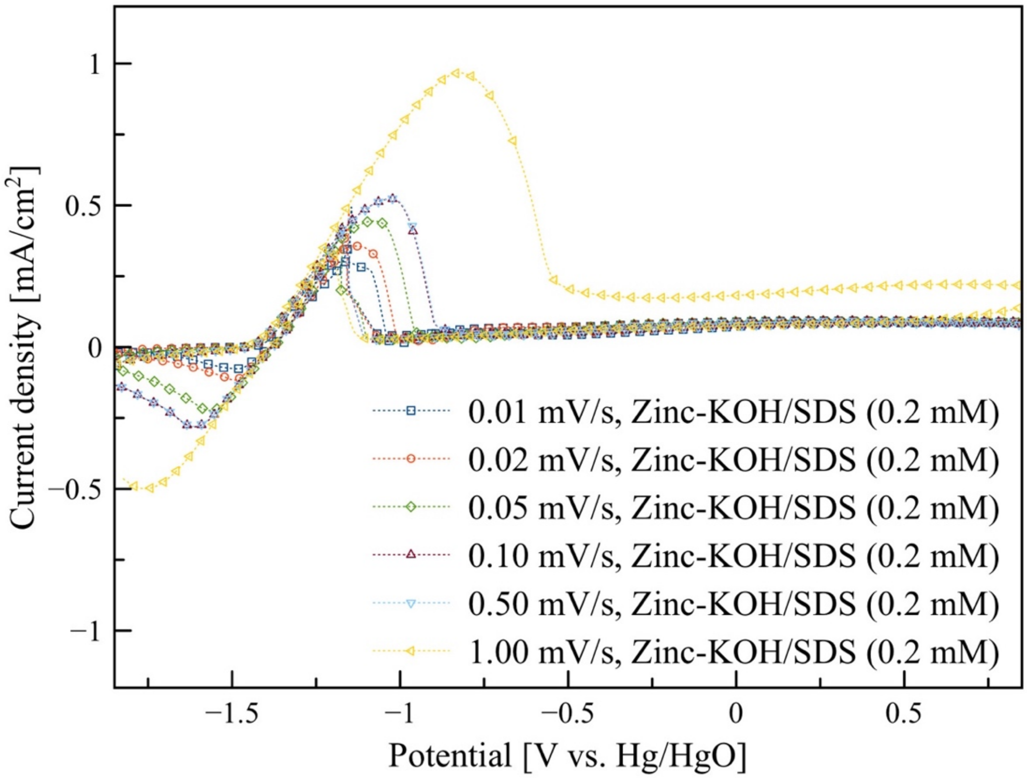

Fig. S2 The effects of scan rate on the voltammetric behavior of zinc plate in the KOH-SDS electrolyte (0.2 mM SDS).

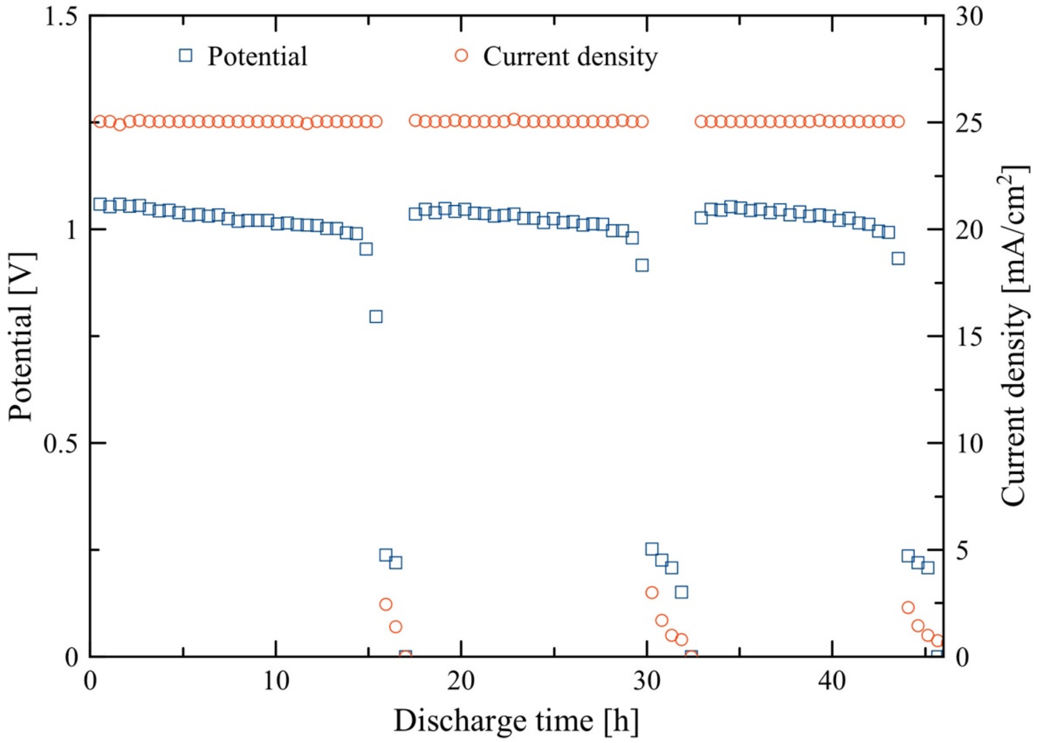

Fig. S3 Multiple galvanostatic discharge profile of the battery using SDS/zinc-KOH (0.2 mM SDS) with the electrolyte circulation rate of 150 mL/min at the discharge current density of 25 mA/cm^2.
